# Supplementary material for: Endothelial Cell Apoptosis but Not Necrosis Is Inhibited by Ischemic Preconditioning
Source: Int J Mol Sci. 2024 Jan 19;25(2):1238. doi: 10.3390/ijms25021238 (PMC10816637; doi:10.3390/ijms25021238)

SUPLEMENTARY MATERIALS

Figure S1. HUVECs stained against von Willebrand factor at baseline (magnification 40x).

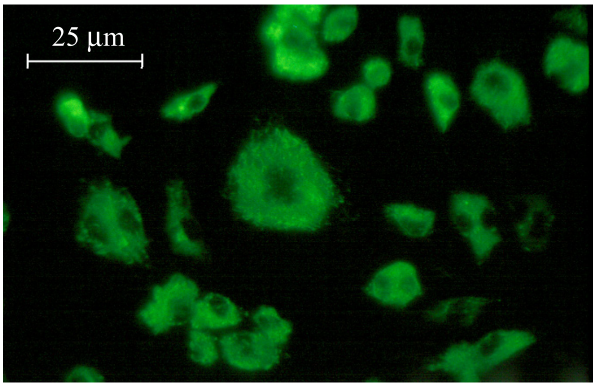

Figure S2. Cell membrane injury of HUVECs following hypoxia and reoxygenation (A - 8 hours, B - 24 hours, magnification 40x). Annexin V labeled with a green, fluorescent dye is attached to the cell membrane of apoptotic cells. The irregular shape of the cell nucleus stained with propidium iodide (B, arrow) indicates DNA fragmentation as a result of apoptosis.

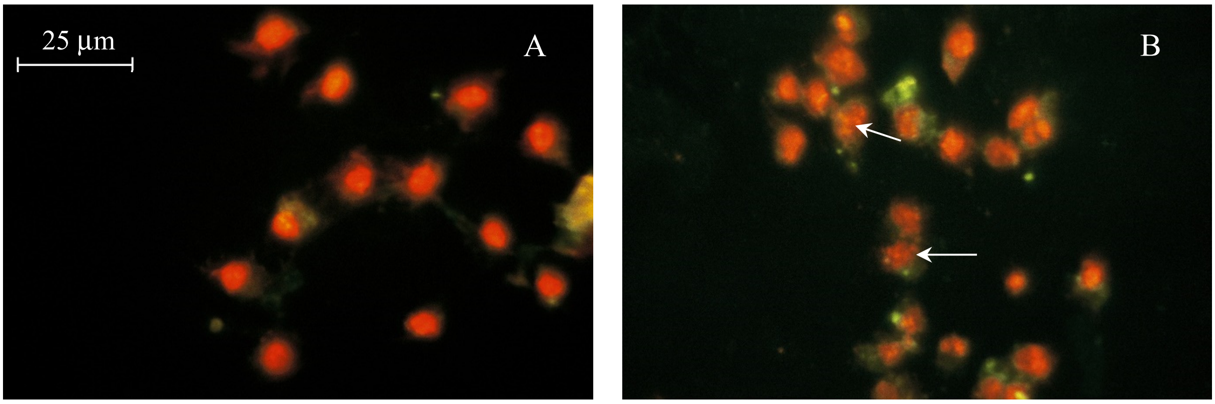

Figure S3. Cell nucleus injury of HUVECs following hypoxia and reoxygenation (24 hours, magnification 100x). (A) TUNEL staining, (B) propidium iodide staining. As a result of endonucleases, nuclear DNA was fragmented, and the so-called sticky ends (arrow) were labeled a green fluorescent dye.

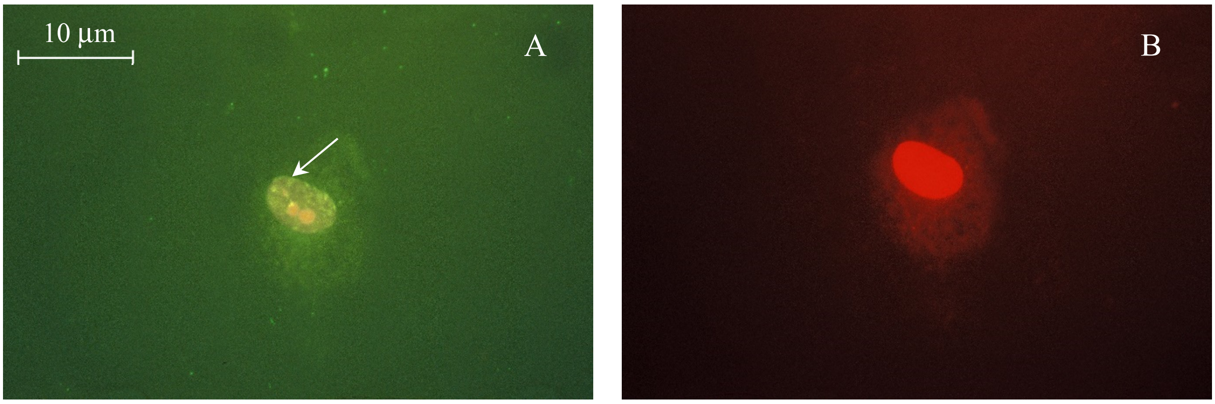

Figure S4. Phase-contrast microscopy of HUVEC (magnification 40x). (A) At baseline. (B) Following hypoxia (2 or 6 hour) there were no changes in HUVEC morphology. Following 8 hours of reoxygenation endothelial cells (C - 2 hours hypoxia, D - 6 hours hypoxia) underwent shrinkage and their shape transformed from polygonal to irregular. As reoxygenation progressed up to 24 hours, increasing intercellular spaces became apparent due to cell shrinkage and detachment from the dish bottom (E - 2 hours hypoxia, F - 6 hours hypoxia)

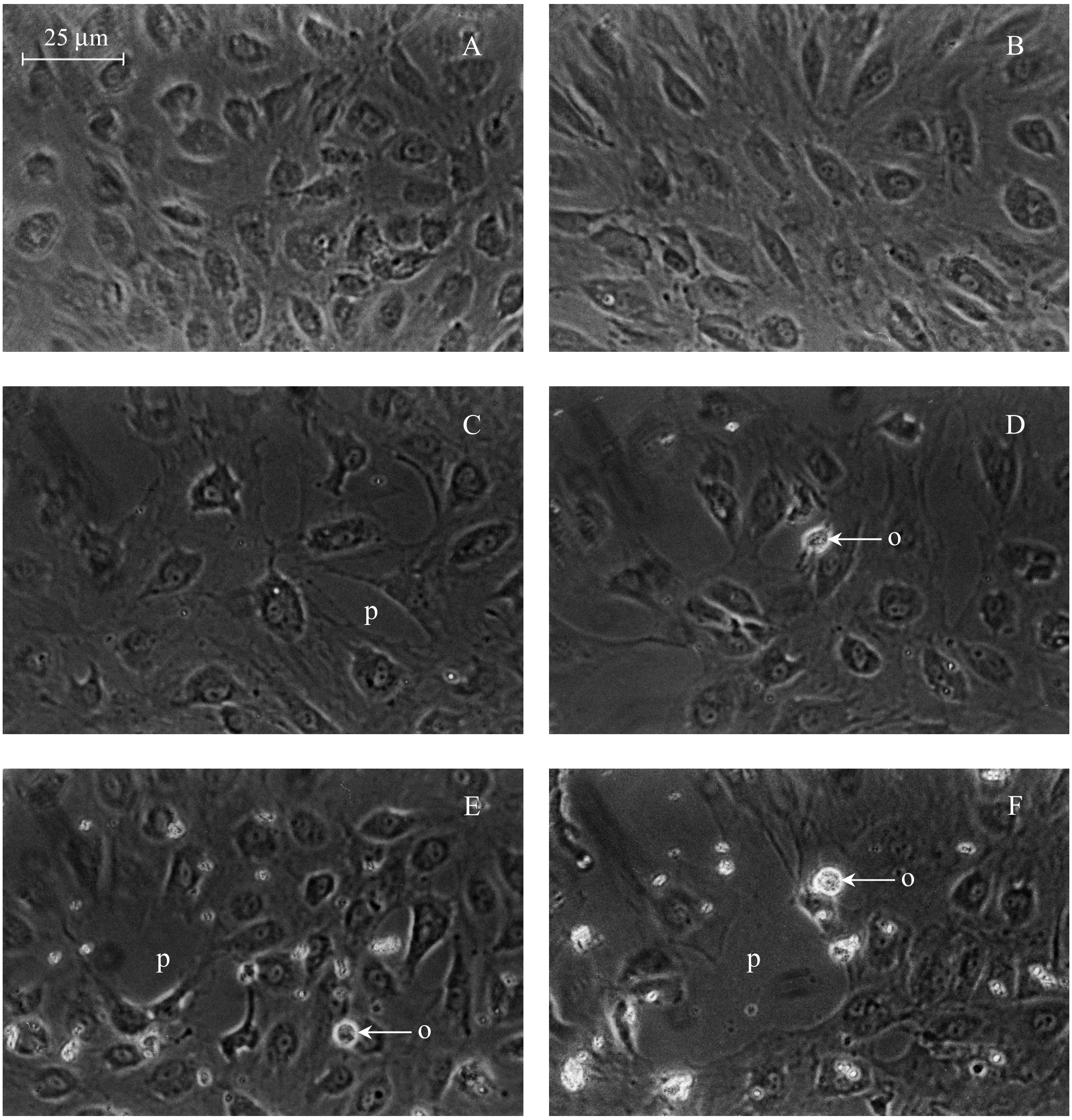

Figure S5. Actin cytoskeleton of HUVEC (magnification 40x). (A) At baseline. (B). Following hypoxia, actin filaments underwent reorganization, and stress fibers were either lost or shortened. After 8 hours of reoxygenation, F-actin accumulated in the perinuclear space, while stress fibers were degraded (C). After 24 hours of reoxygenation, the effects of hypoxia and the early reoxygenation phase were reversed, and stress fibers were rebuilt (D).

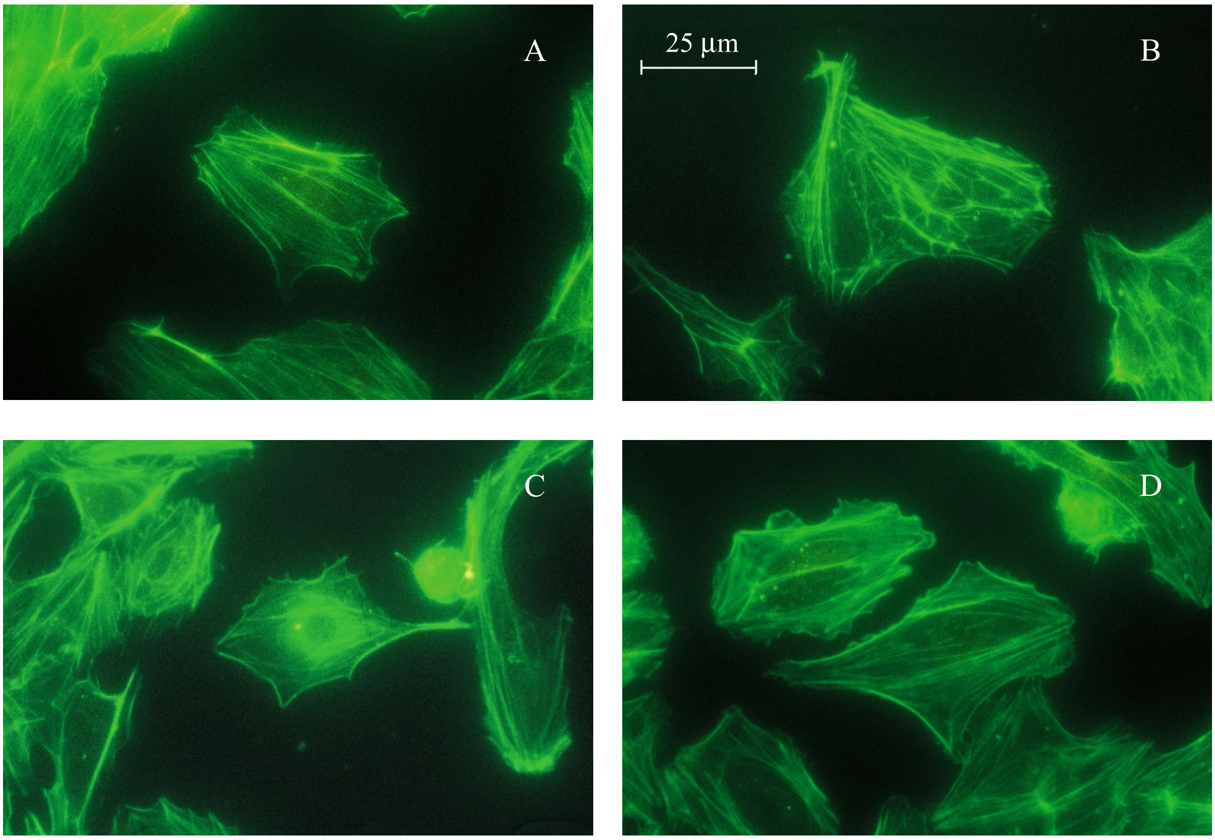

Supplement: Supplementary file 1 [file ijms-25-01238-s001.zip › ijms-2802337-supplementary.pdf]
